# First report of phenotypic and genotypic (bla_OXA-61_) beta-lactam resistance in *Campylobacter jejuni* from broilers in Indonesia

**DOI:** 10.14202/vetworld.2023.2210-2216

**Published:** 2023-11-02

**Authors:** Sheila Marty Yanestria, Mustofa Helmi Effendi, Wiwiek Tyasningsih, Mariyono Mariyono, Emmanuel Nnabuike Ugbo

**Affiliations:** 1 Doctoral Program in Veterinary Science, Faculty of Veterinary Medicine, Universitas Airlangga. Jl. Dr. Ir. H. Soekarno, Kampus C Mulyorejo, Surabaya 60115, East Java, Indonesia; 2 Department of Veterinary Public Health, Faculty of Veterinary Medicine, Universitas Airlangga, Jl. Dr. Ir. H. Soekarno, Kampus C Mulyorejo, Surabaya 60115, East Java, Indonesia; 3 Department of Veterinary Microbiology, Faculty of Veterinary Medicine, Universitas Airlangga, Jl. Dr. Ir. H. Soekarno, Kampus C Mulyorejo, Surabaya 60115, East Java, Indonesia; 4 Bacteriology Laboratory, Balai Besar Veteriner Wates, Jl. Yogyakarta-Wates No. Km. 27, Wates, Yogyakarta 55651, Central Java, Indonesia; 5 Department of Applied Microbiology, Faculty of Science, Ebonyi State University, Enugu - Abakaliki Rd, 481101, Abakaliki, Ebonyi, Nigeria

**Keywords:** antimicrobial resistance, beta-lactam, bla_OXA-61_, *Campylobacter jejuni*, human health

## Abstract

**Background and Aim:**

*Campylobacter* is a zoonotic bacterium that is a major source of foodborne diseases. In humans, most cases of campylobacteriosis are caused by *Campylobacter jejuni*. Poultry is the main reservoir of *Campylobacter* for humans, because *Campylobacter* is part of the normal flora of the digestive tract of poultry. Antimicrobial resistance to several antibiotics in *Campylobacter* isolated from humans and food animals has increased rapidly. Beta-lactam is an antibiotic with a high prevalence of resistance in *Campylobacter*. This study aimed to investigate phenotypic and genotypic (bla_OXA-61_) beta-lactam resistance in *C*. *jejuni* from broilers in Indonesia.

**Materials and Methods:**

A total of 100 samples of broiler intestinal contents were obtained from 10 broiler farms in Pasuruan Regency, Indonesia. *Campylobacter jejuni* was identified using conventional and polymerase chain reaction (PCR)-based methods. Phenotypic detection of beta-lactam resistance was performed using an antimicrobial susceptibility test with antibiotic disks of aztreonam, ampicillin, and amoxicillin-clavulanic acid. Genotypic detection by PCR was performed using the bla_OXA-61_ gene, which encodes beta-lactamase.

**Results:**

*Campylobacter jejuni* was identified in 23% of the samples. Phenotypically, 100% (23/23) and 73.9% (17/23) *C. jejuni* isolates had high resistance to aztreonam and ampicillin, respectively, but all isolates were susceptible to amoxicillin–clavulanic acid. Genotypically, all isolates carried bla_OXA-61_, indicated by the presence of a 372-bp PCR product.

**Conclusion:**

*Campylobacter jejuni* is highly resistant to beta-lactams and is a serious threat to human health. Resistance to beta-lactams should be monitored because beta-lactamase genes can be transferred between bacteria. Public awareness must also be increased on the importance of using antibiotics rationally in humans and animals.

## Introduction

*Campylobacter* is a zoonotic pathogen that is a major source of foodborne bacterial illness worldwide [1]. In humans, campylobacteriosis is caused by *Campylobacter jejuni*, whereas the remainder is mostly caused by *Campylobacter coli* [2]. Recently, the incidence and prevalence of campylobacteriosis have been increasing in both developing and developed countries [1]. According to the Centers for Disease Control and Prevention, campylobacteriosis affects >1.5 million people (excluding undiagnosed and unreported cases) in the United States every year [3]. Campylobacteriosis is generally self-limiting with fever, stomach cramps, and bloody diarrhea. However, campylobacteriosis can become chronic and cause reactive arthritis, Guillain-Barré syndrome, Miller-Fisher syndrome, urinary tract infection, irritable bowel, sepsis, and certain neuropathies. [4].

Poultry is the main source of *Campylobacter* transmission to humans, because it is a part of the normal flora in the digestive tract of poultry [5]. *Campylobacter* spp., especially *C. jejuni*, can be found in high numbers in ceca broilers at the farmer level and on carcasses in poultry slaughterhouses and meat markets [6]. *Campylobacter* can be transmitted from poultry through activities that may expose humans to poultry excrement, or by handling and consuming contaminated meat [7]. In Indonesia, 61.9% of chicken meat was reported to contain *Campylobacter* and most isolates (41.07%) were *C*. *jejuni* [8]. In South Asia, the incidence of antimicrobial resistance in *Campylobacter* isolated from humans and food animals has increased during the past decade, especially in countries with widespread use of antibiotics in livestock [9–11]. *Campylobacter* spp. are highly resistant to beta-lactam antibiotics [11]. *Campylobacter* strains that produce beta-lactamase were more resistant to ticarcillin, amoxicillin, and ampicillin than beta-lactamase-negative strains. This is attributed to the bla_OXA-61_ gene, which encodes Cj0299-a putative periplasmic amino acid class D beta-lactamase [12]. The overuse of antibiotics in human and animal populations has increased the number of antimicrobial-resistant bacteria [13]. Antimicrobial resistance poses an additional risk because infection with antibiotic-resistant *Campylobacter* leads to longer hospitalizations, higher rates of treatment failure, and increased morbidity and mortality [14].

In Indonesia, information regarding antimicrobial resistance in *C*. *jejuni* is limited; therefore, this study aimed to investigate phenotypic and genotypic (bla_OXA-61_) beta-lactam resistance in *C*. *jejuni* from broilers. An antimicrobial susceptibility test was used for phenotype detection, and polymerase chain reaction (PCR) for bla_OXA-61_ was used for genotype detection.

## Materials and Methods

### Ethical approval

Animal ethical approval was obtained from the Ethical Clearance Committee of the Faculty of Veterinary Medicine, Universitas Wijaya Kusuma Surabaya, Indonesia (ethics no.: 86-KKE/2022).

### Study period and location

The study was conducted from October 2022 to December 2022 at the Balai Besar Veteriner Wates Yogyakarta and the Institute of Tropical Disease Airlangga University.

### Sample collection

A total of 100 samples of broiler intestinal contents were obtained from ten broiler farms in Pasuruan Regency, Indonesia. Sampling was performed by slaughtering broilers and dissecting the abdomen to obtain the contents of the small intestine. Samples were placed in sterile plastic and stored in a cool box on the way to the laboratory.

### Isolation and identification of *C. jejuni* strains

#### Bacterial enrichment

The sample was placed in a dark bottle containing 40 mL of Bolton Broth (Oxoid AM7526, England), supplemented with 5% lysed sheep blood, Preston supplements (Oxoid SR0117, England), ferrous sulfate, sodium metabisulfite, and sodium pyruvate. The sample was incubated at 37°C for 4 h and then at 42°C for 24 h under microaerophilic conditions (5% O_2_, 10% CO_2_, 85% N_2_) [15].

#### Isolation and identification

*Campylobacter* isolation was performed using a modified BAM 2001 method [15]. One loop pellet was streaked on modified charcoal cefoperazone deoxycholate selective medium containing charcoal cefoperazone deoxycholate selective supplement (Oxoid SR 155E, England) and incubated at 42°C for 48 h under microaerophilic conditions. Isolates were confirmed as *Campylobacter* by microscopic examination, Gram staining, and biochemical tests for catalase and oxidase.

### Genotypic detection of *C. jejuni* strains (hipO gene) and beta-lactam resistance (bla_OXA-61_ gene)

#### DNA extraction

QIAamp DNA Mini Kit (Qiagen, Hilden, Germany) and QIAprep Spin Miniprep Kit (Qiagen) were used to extract genomic and plasmid DNA, respectively, according to the manufacturer’s recommendations.

#### Oligonucleotides primers

Polymerase chain reaction primers were synthesized by Integrated DNA Technologies (Iowa) (Table-1) [16, 17].

**Table-1:** Primer sequences, target genes, amplicon sizes, and cycling conditions.

Target gene	Primer Sequences	Size (bp)	Primary denaturation	Amplification (35 cycles)			Final Extension	Reference

				Secondary denaturation	Annealing	Extension		
hipO	F-ACTTCTTTATTGCTTGCTGC	323	95°C	95°C	59°C	72°C	72°C	[16]
	R-GCCACAACAAGTAAAGAAGC		0.5 min	0.5 min	0.5 min	0.5 min	7 min	
bla_OXA-61_	F-AGAGTATAATACAAGCG	372	95°C	95°C	54°C	72°C	72°C	[17]
	R-TAGTGAGTTGTCAAGCC		5 min	50 s	30 s	1 min	7 min	

#### Polymerase chain reaction assay

The PCR master mix contained 5 μL DNA template, 1 μL each primer, 0.5 μL nuclease-free water, and 12.5 μL PCR master mix (Promega, USA), containing Taq DNA polymerase, dNTPs, MgCl_2_, and reaction buffer. The final volume of the reaction mixture was 20 μL.

The PCR products were separated by electrophoresis in 1.5% agarose gel (Invitrogen, USA), supplemented with RedSafe Nucleic Acid Staining Solution gel dye (Intron, South Korea). The size of the PCR products was evaluated using a 100-bp DNA marker. The gel was electrophoresed for 30 min at a constant voltage of 100 V and visualized under ultraviolet light.

### Phenotypic detection of beta-lactam resistance using an antibiotic sensitivity test

All *C*. *jejuni* isolates were analyzed for antimicrobial resistance on Mueller–Hinton agar plates (Oxoid CM 0337b, England), supplemented with 5% defibrinated sheep blood, containing ampicillin (10 μg), aztreonam (30 μg), and amoxicillin–clavulanic acid (30 μg) disks. The medium was incubated at 42°C for 24 h under microaerophilic conditions [18]. The bacterial susceptibility to antibiotics was interpreted by measuring the inhibition zone and using the Clinical Laboratory Standard Institute table as a standard [19].

## Results and Discussion

In this study, samples were collected from the small intestine, especially from parts of the jejunum, because the results for *C*. *jejuni* isolation were good. *Campylobacter jejuni* mainly colonizes the ceca and small intestine [20]. *Campylobacter jejuni* can colonize the intestinal tract, especially at the junction between the ceca, small intestine (jejunum), and large intestine, where their number is high (≥10^8^ Colony-forming unit/g) [21].

**Figure-1:**
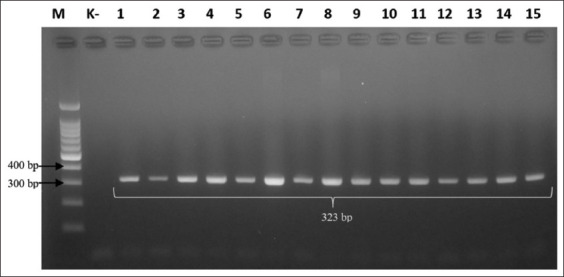
Gel electrophoresis results for detection of *Campylobacter jejuni* using the hipO gene (323-bp). Note: Lanes: M, 100-bp marker; K-, control negative; 1-15, representative of *Campylobacter jejuni* isolates.

Based on the results of isolation, morphological identification, and biochemical tests (catalase and oxidase tests), *Campylobacter* was detected in 44/100 samples. Polymerase chain reaction analysis showed that 23% (23/100) isolates were positive for *C*. *jejuni* (323-bp product) (Table-2 and Figure-1). Moreover, 52.3% (23/44) *Campylobacter* isolates were *C*. *jejuni*, which is most commonly found in poultry, whereas *C. coli* is predominant in pigs [12].

**Table-2:** Results of isolation, identification, and PCR test to detect *Campylobacter jejuni.*

Total samples	Isolation (culture on mCDDA)		Identification (microscopy, Gram stain, biochemical tests)		PCR test hipO gene	
		
	No of positive		No of positive		No of positive	
100	No	%	No	%	No	%
	44	44	44	44	23	23

In Indonesia, the prevalence of *C*. *jejuni* in poultry is 23%. The prevalence rates were similar to our study in Jordan 17% [22], Ecuador 18.9% [23], Benin (West Africa) 23.4% [24], and India 24% [25]. Polymerase chain reaction after selective enrichment is recommended for identifying *Campylobacter* in drinking water, dairy products, aquatic habitats, and poultry products. In other countries, prevalence rates vary, which is probably influenced by variations in sampling design and testing technique [25].

**Figure-2:**
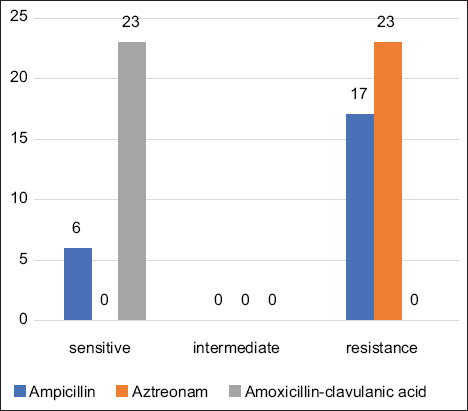
Results of the antimicrobial susceptibility test of *Campylobacter jejuni* isolate.

Based on antimicrobial susceptibility tests, *C*. *jejuni* isolates had the highest level of resistance to aztreonam 100% (23/23) and a lower level of resistance to ampicillin 73.9% (17/23). However, all isolates (23/23) were susceptible to amoxicillin-clavulanic acid (Figure-2).

In this study, all *C*. *jejuni* isolates were resistant to aztreonam (100%), which is consistent with results from Lebanon [12] and Jordan [26]. Most *C*. *jejuni* are resistant to ampicillin (74%), which is the most commonly used antibiotic in poultry [27]. This result is similar to studies in South China 70.7% [28], but lower than the incidence in several countries with a range of 81.8%–100% [26, 29, 30]. The combination of amoxicillin-clavulanic acid is the most efficient against *C*. *jejuni* based on the results of this study, because all *C*. *jejuni* isolates were susceptible to amoxicillin-clavulanic acid. These results are similar to studies in the Lebanon [12] and United Kingdom [31]. In contrast, a study in Tunisia showed that *C*. *jejuni* was 22.7% resistant to amoxicillin-clavulanic acid [29].

**Figure-3:**
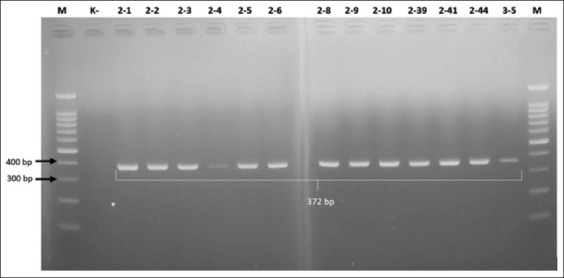
Gel electrophoresis results for detection of beta-lactam resistance using the bla_OXA-61_ gene in *Campylobacter jejuni* isolate (372-bp). Note: Lanes: M, 100-bp marker; K-, control negative; 2-1 – 3-5, representative of *Campylobacter jejuni* isolates.

This study focuses on discovering the gene (bla_OXA-61_) in *C. jejuni*, a new gene discovered in Indonesia. Polymerase chain reaction detected the bla_OXA-61_ gene (372-bp PCR product) in all 23 *C*. *jejuni* isolates (Figure-3). Six *C*. *jejuni* isolates that were susceptible to ampicillin also carried the bla_OXA-61_ gene. Most *C*. *jejuni* strains can produce beta-lactamase, which inactivates beta-lactam by hydrolyzing the lactam ring [32]. Beta-lactamase is divided into four classes according to Ambler’s classification: class A (KPC and most extended-spectrum β-lactamase), class B (MBL), class C (AmpC beta-lactamase), and class D (OXA). The bla_OXA-61_ gene belongs to class D and is widely encoded in integrons [33]. Class D beta-lactamase OXA-61 is the most frequently identified in *Campylobacter* [34].

In this study, all *C*. *jejuni* isolates were phenotypically resistant to aztreonam and genotypically possessed the bla_OXA-61_ gene, indicating a strong correlation between the two. Consistently, class D beta-lactamase, which consists only of OXA, can hydrolyze cephalosporins and aztreonam and has carbapenemase activity [35]. The ability to hydrolyze monobactam (aztreonam) is shared by class A and class C, but not class B, beta-lactamases [33, 36].

Molecular investigation of antimicrobial resistance in the tested isolates showed a strong correlation between the antibiotic resistance phenotype and the genotypes and mutations coding for antibiotic resistance [18]. In this study, all isolates that were phenotypically resistant to ampicillin were detected by the bla_OXA-61_ gene, which encodes antimicrobial resistance to beta-lactams. Most ampicillin-resistant *Campylobacter* isolates from poultry carried the bla_OXA-61_ gene, according to other studies conducted in the UK [31] and Brazil [37].

However, despite the strong correlation between antimicrobial resistance to beta-lactams and bla_OXA-61_, 6 ampicillin-susceptible *C*. *jejuni* isolates possessed this gene. Our results are supported by a report in which 59% of ampicillin-susceptible isolates carried bla_OXA-61_ [38], indicating that bla_OXA-61_ is poorly expressed in ampicillin-susceptible isolates, and therefore, less beta-lactamase is produced compared with resistant isolates [39]. This could be because there is a relationship between ampicillin susceptibility and the presence of G-T transversion in the bla_OXA-61_ promoter, only isolates that are resistant to ampicillin have G-T transversion [38]. Alfredson and Korolik [34] (GenBank accession number AY587956) identified bla_OXA-61_ in ampicillin-resistant *C*. *jejuni* isolates with G-T transversion. A large-scale study is warranted to evaluate the association between G-T transversion and high-level bla_OXA-61_-mediated beta-lactam resistance in *C. jejuni* isolates [38].

CmeABC efflux pumps contribute to beta-lactamase resistance and could explain the ampicillin susceptibility of *C*. *jejuni* strains carrying bla_OXA-61_ [12]. In a study, ampicillin susceptibility increased 32-fold after CmeB insertion mutagenesis in *C*. *jejuni* strain 81-176136 and other strains [40]. In another study utilizing the NCTC 11168 strain, the CmeB mutant was four times more susceptible to ampicillin than parental strain, and cmeB overexpression increased ampicillin resistance four folds [41].

In this study, all *C*. *jejuni* isolates susceptible to amoxicillin–clavulanic acid carried bla_OXA-61_, indicating that this combination strongly inhibits beta-lactamase and can be used as an alternative to fluoroquinolone, tetracycline, and erythromycin, which *C*. *jejuni* is resistant to [31, 39]. In *C*. *jejuni*, the expression of beta-lactamase, which causes resistance to amoxicillin, ampicillin, and ticarcillin, can be inhibited with tazobactam, clavulanic acid, and sulbactam [12].

Antimicrobial resistance in pathogenic microorganisms, especially *C*. *jejuni*, is a global health challenge. According to the Centers for Disease Control and Prevention, drug-resistant strains of *Campylobacter* infect >300,000 individuals each year, making treatment a major health challenge and economic burden. Antimicrobial resistance in bacteria can be generated by antibiotic overuse by humans, antibiotic use in animal feed or veterinary treatment, and increased industrial waste in the environment [26, 32, 42]. Bacterial resistance to antimicrobials has evolved in a variety of ways; in most situations, bacteria exposed to antibiotics discover ways to avoid or resist antimicrobial agents [31, 40, 43, 44].

## Conclusion

Our results show that *C*. *jejuni* has high resistance to beta-lactams and is a serious threat to human health. Beta-lactam resistance should be monitored because beta-lactamase genes can be transferred between bacteria. Public awareness of the importance of using antibiotics rationally in humans and animals must be increased. More studies are needed to understand antibiotic resistance to and develop diagnostic media for *Campylobacter* spp. To the best of our knowledge, this is the first study to genotypically identify beta-lactam resistance in C. *jejuni* from Indonesian poultry. Our findings highlight the importance of a surveillance and monitoring system and risk analysis for C. *jejuni* prevalence and resistance in poultry and other animal feeds.

## Authors’ Contributions

SMY, MHE, and WT: Conceptualized and supervised the study and drafted the manuscript. SMY: Data curation and formal analysis. SMY and MM: Investigation and visualization. MHE and WT: Methodology. MHE, WT, and ENU: Validation. WT and ENU: Review and editing. All authors have read, reviewed, and approved the final manuscript.
